# Ocular Complications after COVID-19 Vaccination: A Systematic Review

**DOI:** 10.3390/medicina60020249

**Published:** 2024-01-31

**Authors:** Eman A. Hurissi, Ismail I. Abuallut, Mohammed Qassem Dibaji, Abdulaziz Jaly, Abdulaziz H. Alhazmi, Bandar M. Abuageelah, Khalid M. Alameer, Yousef M. Alyami

**Affiliations:** 1 Faculty of Medicine, Jazan University, Jazan 45142, Saudi Arabia; ehurissi@moh.gov.sa (E.A.H.); 202101533@stu.jazanu.edu.sa (K.M.A.); 2 Department of Surgery, Ophthalmology Division, Jazan University, Jazan 45142, Saudi Arabia; iabuallut@jazanu.edu.sa; 3 Prince Mohammed Bin Naser Hospital, Jazan 82943, Saudi Arabia; mdibaji@moh.gov.sa; 4 Pharmacy, Jazan University Hospital, Jazan 45142, Saudi Arabia; ajali@jazanu.edu.sa; 5 Department of Medicine and Surgery, Batterjee Medical College, Abha 62451, Saudi Arabia; 21210064@bmc.edu.sa (B.M.A.); 21210184@bmc.edu.sa (Y.M.A.)

**Keywords:** COVID-19, complications, ocular, vaccines

## Abstract

*Background and Objectives*: The COVID-19 pandemic affects various populations worldwide. The discovery of vaccinations was necessary for the prevention and elimination of the disease. Despite the high importance of these vaccinations, they may cause some complications, such as ocular complications. This study aims to draw attention to the possible complications of the vaccination and highlight its importance. *Materials and Methods*: Systematic review of the literature from January 2021 to January 2023. A total of 20 published articles were included and reported cases of ocular complications in patients who received COVID-19 vaccines. *Results*: A total of 243 patients with verified ocular complications following the COVID-19 vaccination were included, ranging in age from 18 to 84 years. The most common ocular complications reported in the current study were ocular inflammatory complications, which represented 47.3%, followed by optic neuritis (24.3%). Retinal artery occlusion, retinal vein occlusion, acute macular neuroretinopathy, and paracentral acute middle maculopathy represented 10.7%. Herpetic ocular infections and herpetic eye disease (14%). Nearly half (42%) of the patients with ocular problems received the Pfizer-BioNTech vaccination. *Conclusions*: Despite the high importance of the COVID-19 vaccination, it was found that it is associated with the occurrence of some ocular complications. Future projects should come with more extensive prospective studies to further elucidate the underlying mechanisms and risk factors associated with ocular complications following COVID-19 vaccination, thereby enhancing our understanding and guiding appropriate management strategies.

## 1. Background

The international scientific community has been compelled by the serious COVID-19 pandemic to find treatments and vaccines to control the SARS-CoV-2 virus. On 11 January 2020, the genetic makeup of SARS-CoV-2, the coronavirus that causes COVID-19, was made public. This sparked a surge in global research and development efforts to create a vaccine to prevent this dangerous illness [1]. The COVID-19 vaccination is considered a critical tool for preventing this pandemic and one of the most effective strategies for eliminating it [2]. Different vaccines have been developed in an effort to reduce the morbidity and mortality caused by COVID-19 and stop viral transmission. The U.S. Food and Drug Administration (FDA) authorized the first COVID-19 vaccination, also known as the Pfizer-BioNTech (Pfizer, New York, NY, USA) COVID-19 Vaccine, for emergency use in December 2020. Since then, additional vaccines have been authorized for the prevention of COVID-19 disease that utilize vectors (Ad26.COV2, Janssen Johnson & Johnson; ChAdOx1 nCoV-19/AZD1222, Oxford-AstraZeneca, Oxford, UK), mRNA (mRNA-1273, Moderna, Cambridge, MA, USA), and protein subunits (NVXCoV2373, Novavax, Gaithersburg, MD, USA) [3].

Despite the urgent need and extreme importance of vaccines, which save millions of lives annually, some side effects have been widely reported in many cases. Vaccines are known to promote the development of immunity by inducing immunological responses. Still, they also carry the danger of immune-mediated adverse effects on all human body areas [4]. Little is currently known about potential post-vaccination ocular-specific consequences. Therefore, scientists and eye care medical professionals must look into any possible adverse effects of COVID-19. Previous studies have shown a connection between COVID-19 infection and direct or indirect ocular problems. It is well known that COVID-19 infection can cause conjunctivitis, scleritis, orbital inflammatory illness, phlyctenular keratoconjunctivitis, and retinal involvement [5]. In the current study, we aimed to review and discuss all reported ocular complications following COVID-19 vaccinations.

## 2. Material and Methods

### Research Strategy

We extensively examined the following databases: PubMed, Embase, Medline, Cochrane Central, and Google Scholar, for research on related themes, in order to fully determine the ocular side effects of the COVID-19 vaccination. The literature review covers the period between January 2021 and January 2023.

The following search terms were used to access the databases: (“2019-nCoV” or “nCoV-19” or “nCoV-2019” or “SARS-CoV-2” or “SARS-CoV2” or “SARS2” or “COVID” or “coronavirus” or “coronavirus disease 2019” or “severe acute respiratory syndrome” vaccine) and (“eye” or “ocular” or “oculopathy” or “oculomotor” or “intraocular” or “Ophthalmic”).

Additionally, we looked for other relevant publications in the included studies’ references. The literature review was completed in January 2023. Original articles on ocular complications experienced by COVID-19 vaccination recipients met the inclusion criteria. The case reports were also included in the current study, while studies without any data on ocular complications experienced by patients who received the COVID-19 vaccination were excluded.

We included all patients, regardless of age and sex, who reported experiencing ocular complications after receiving a COVID-19 vaccination.

**Figure 1:**
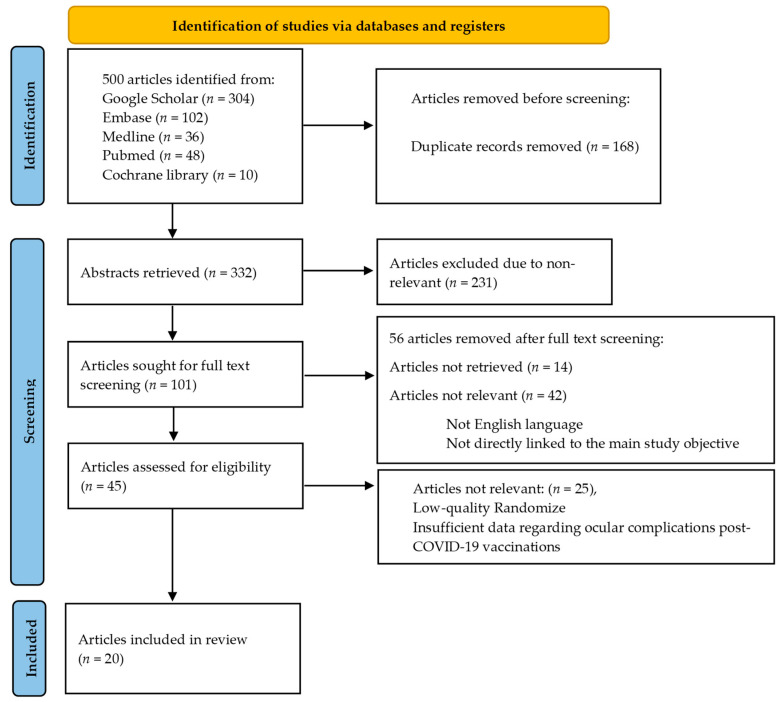
Shows a flowchart that illustrates the steps involved in retrieving articles.

We reviewed 332 abstracts to determine their relevance to ocular complications that may occur after receiving the COVID-19 vaccine. Two authors independently assessed the abstracts and resolved any discrepancies through discussion. We excluded abstracts that were not relevant. Of the remaining articles, 101 were screened in full text, and the authors resolved any differences. Articles that did not have any data regarding ocular complications in COVID-19 vaccine recipients were excluded. Ultimately, 20 articles met our inclusion criteria and were included in our analysis (Figure 1).

## 3. Results

Two hundred forty-three patients with verified ocular complications after receiving the COVID-19 vaccine were assessed across 20 studies from January 2021 to January 2023 (Table 1). A total of 243 cases, ranging in age from 18 to 84 years, with a median age of 50–65 years, with ocular complications post-COVID-19 vaccination, were included in this study. The most commonly reported ocular potential complications were higher in females across all age categories. The most common ocular complications reported in the current study were ocular inflammatory complications, representing 115 patients (47.3%). It includes “uveitis, anterior and posterior uveitis, scleritis, posterior scleritis, choroiditis, keratitis, acute retinal necrosis, and iridocyclitis”. “Optic neuritis” represented 59 patients (24.3%). Herpetic ocular infections and herpetic eye disease represented 34 patients (14%), while retinal artery occlusion, retinal vein occlusion, acute macular neuroretinopathy, and paracentral acute middle maculopathy represented 26 patients (10.7%) (Table 2).

The adverse ocular complications were categorized according to the vaccine manufacturer/name to identify the vaccination type linked to these ocular problems (Table 3). Around 102 (42%) of the patients with ocular adverse events received the Pfizer-BioNTech vaccination, and 69 (28.3%) of the cases received the Astra-Zeneca vaccine.

In the current study, it can also be observed that the ocular complications, which include “Uveitis, Anterior uveitis, posterior uveitis, scleritis, choroiditis, keratitis, acute retinal necrosis, and iridocyclitis”, are highly associated with Pfizer vaccines (54.8%), while in relation to “optic neuritis”, they are highly associated with AstraZeneca vaccines (69.5%). Also, it is highly associated with mRNA and adenoviral vector vaccines concerning retinal artery occlusion, retinal vein occlusion, acute macular neuroretinopathy, and paracentral acute middle maculopathy.

## 4. Discussion

The COVID-19 pandemic has profoundly impacted human health and the global economy. The advent of COVID-19 vaccines, accompanied by regulatory approval and widespread administration worldwide, marks a hopeful milestone in combating the pandemic. On 11 December 2020, the FDA granted emergency use authorization for COVID-19 vaccines, underscoring the significance of vaccines as one of medicine’s most significant achievements [1]. Despite their relatively rare occurrence, this study aimed to assess ocular complications following the COVID-19 vaccination comprehensively. Nevertheless, a diverse range of ocular complications was identified among individuals who received COVID-19 vaccines.

The most prevalent ocular complications reported in this study were classified as “ocular inflammatory complications”, encompassing uveitis, anterior uveitis, posterior uveitis, scleritis, posterior scleritis, choroiditis, keratitis, acute retinal necrosis, and iridocyclitis, collectively accounting for 47.3% of all cases. These findings align with previous literature reviews [26,27], which identified uveitis as the most common ocular complication after receiving vaccines for various conditions. Notably, our study’s predominant subtype of uveitis observed was anterior uveitis, representing 56% of cases. Furthermore, another study also reported scleritis as a common ocular complication following the COVID-19 vaccination, corroborating our findings [28].

Optic neuritis emerged as our investigation’s second-most frequent ocular complication, accounting for approximately 24.3% of cases. This result concurs with prior research [29] linking COVID-19 immunization to various types of optic neuropathy, particularly ischemic optic neuropathy and optic neuritis. Additionally, optic neuropathy was associated with all COVID-19 vaccine subtypes, including mRNA, viral vector, and inactivated viral vaccines. Moreover, a comprehensive review of adverse ocular events from 2010 to 2020 by Cheng and Margo [30] found optic neuritis to be the most frequently reported ocular adverse event across nine distinct vaccines. The exact mechanisms underlying vaccine-induced optic neuritis remain unclear. Still, previous research has proposed the possibility of molecular resemblance between viral proteins and myelin basic proteins and the involvement of epitope dissemination, bystander activation, and superantigen activation [5].

Regarding herpetic ocular infections, which are severe complications and a significant cause of infectious blindness [31], the present study identified them as the third most common ocular complication following COVID-19 vaccinations, accounting for approximately 14% of cases. This observation is consistent with Cohen et al.’s review [15], reporting the occurrence of herpetic ocular infections following SARS-CoV-2 vaccinations. Retinal vascular occlusion was identified as another common ocular complication, aligning with Sonawane, Yadav, Kota, and Singh’s study [32], which reported the development of central retinal vascular complications following COVID-19 vaccinations. Remarkably, all types of COVID-19 vaccines were associated with ocular complications, with the data suggesting a higher frequency of ocular complications among recipients of Pfizer vaccines. This finding is consistent with a literature review by Sonawane et al. [32].

Concerning peripheral ulcerative keratitis, the current review mentioned a single case of post-COVID-19 vaccine-complication peripheral ulcerative keratitis. Our result is consistent with the study of Kuziez et al., who report that the occurrence of herpetic keratitis is common after SARS-CoV-2 vaccination in more than half of all patients [33].

Marginal keratitis as a post-COVID-19 vaccine complication was also reported in one case of our review by Farrell et al. [22]. Approximately 2.5 weeks after receiving the mRNA-1273 vaccine’s initial dosage, a 68-year-old woman complained of increased right eye pain and redness. When her symptoms first appeared, she treated herself with antibiotic eye drops, but nothing changed. Infiltrates of the cornea and peripheral corneal vascularization were present in the right eye during examination, along with a large conjunctival injection. Anomalies of the anterior chamber, discharge, or epithelial defects were nonexistent. It was unimpressive in the left eye. She was prescribed topical anti-biotics and corticosteroids after being found to have marginal keratitis of the right eye, and within a few days, her condition had improved. Concerning the Corneal graft rejection cases, A common and successful solid organ transplant is corneal transplantation [7]. Despite the fact that rejection after immunization is generally thought to be infrequent, the occurrence probably goes unreported. Cornea is considered one of the few tissues in the body with immunological privilege. Immune system entry is blocked by the cornea’s distinct avascular structure and lack of lymphatic tissue. Major Histocompatibility Complexes (MHC) I and II are also expressed at low levels in the corneal layers, which restricts the immune response to antigens. Tregs play a significant part in the downregulation of immunological responses in the cornea. Forkhead box protein 3 (Foxp3), cytotoxic T lymphocyte antigen-4, programmed cell death ligand-1, interleu-kin-10, and transforming growth factor are expressed on the surface of these cells and suppress immune activation by preventing the activity of antigen-presenting cells, CD4+ T [34].

Dendritic cells are present in both the central and peripheral corneas, but because an interleukin-1 receptor antagonist is expressed in the cornea, they are repressed, further excluding the cornea from immune surveillance. The survival of corneal allografts is supported by these pathways and others. It has been speculated that immune system activation and dysregulation following vaccination may put these defenses in jeopardy and expose the immune system to the corneal graft and foreign antigens, triggering rejection [35,36]. A mechanism for acute rejection after SARS-CoV-2 vaccination has been hypothesized, and it involves cross-reactivity between the SARS-CoV-2 antigen and MHC-antigen complexes. The target antigen for the humoral immune response, the spike protein, is encoded by the mRNA molecules BNT162b2 and mRNA-1723, which are lipid-encapsulated. Anti-spike protein titers increase following vaccination. Currently, antibodies that cross-react with molecules from corneal graft donors may trigger an immunological response, resulting in rejection [37].

Based on the corneal responses that have been seen in inflammatory stress situa-tions, another mechanism has been hypothesized. Corneal epithelial cells and dendritic cells express MHC class II and co-stimulatory molecules in response to stress. Such in-flammatory stress may be brought on by the display of donor antigens following im-munization and result in allosensitization [34].

Similar to this, it has been shown that inflammation inside the host bed reduces Foxp3 expression in Tregs and obstructs Treg differentiation, potentially reducing the numerous mechanisms for immunological regulation by Tregs. Additionally, SARS-CoV-2 vaccines induce potent humoral and cellular immunological reactions, including a Th1-biased CD4+ response, as found with previous vaccines. As mediators of corneal transplant rejection, CD4+ Th1 cells might be involved in this situation [38,39].

An immunological response to vaccination adjuvants, which are used to boost the body’s immune response and reduce the frequency and dosage of vaccines required to achieve effective preventive immunity, may be another potential mechanism [16].

At least 34 anecdotal keratoplasty rejection episodes linked to vaccines were dis-covered in a survey of cornea specialists in 2021, but that same study also noted that over the previous 30 years, only four articles describing a total of 12 cases of an association between recent vaccination and corneal transplant rejection had been published [40,41].

Our systematic review is consistent with Shah et al., who report that during the mRNA-1273 vaccination of patients. Four patients who got the vaccination showed signs of rejection two weeks after administration, as described in their case series [42].

Lee and Han described an example of bilateral corneal edema six days after receiving the COVID-19 vaccine; a 55-year-old woman who had undergone successful cataract surgery two months earlier came with an abrupt visual disruption and ocular pain. Her initial 20/20 vision had deteriorated to bilateral 20/30 vision at the time of presentation. A slit-lamp examination indicated mild bilateral corneal edema with a CCT of 580 m and 594 m, respectively, for the right and left eyes. The right eye’s endothelial cell density was 2849/mm^2^, while the left eye’s was 2778/mm^2^. Her vision improved to 20/25 bilaterally after two weeks of topical prednisolone therapy, and the corneal edema in the right eye had disappeared with just minor residual edema in the left eye. Her CCTs also improved to 553 m and 579 m in the right and left eyes, respectively [23].

Despite these significant findings, it is essential to acknowledge the limitations of this study. The relatively small sample size of 243 cases from 20 studies may not fully represent the entire vaccinated population, warranting more extensive and diverse samples to derive robust conclusions. Additionally, this study primarily focused on short-term complications, necessitating longer-term follow-up to detect any delayed effects that may emerge over time. Our analysis observed a higher frequency of complications in females across all age categories. However, the exact reasons for this discrepancy require further investigation. Other limitations include the potential for biases in the included studies, such as selection, reporting, and publication biases, which could impact the validity of the overall findings. Furthermore, the heterogeneity of the included studies in terms of design, participants, vaccine types, and complication definitions poses challenges to comparability and generalizability. Moreover, the lack of a control group in most studies hampers the establishment of a direct causal link between vaccination and ocular complications, potentially confounding this study’s outcomes. Reporting inconsistencies in the studies may also lead to incomplete or unreliable data, further affecting this study’s accuracy. The reliance on temporal association to identify ocular complications after vaccination raises concerns about causality, as other factors or pre-existing conditions may influence the outcomes. The variability in COVID-19 vaccine types, including mRNA, viral vector, and inactivated virus, might also introduce confounding variables in the analysis. Additionally, the absence of specific details regarding vaccine dosages from patients experiencing complications could influence the likelihood and severity of ocular adverse events.

## 5. Conclusions and Recommendations

The invention of the COVID-19 vaccination is one of the most important measures to limit the spread of those dangerous epidemics. Still, it may result in some side effects or complications that can be remedied and dealt with. Overall, the advantages of receiving the COVID-19 vaccine for all populations outweigh the risks of ocular problems that may occur considerably. These uncommon but potential ocular complications following the COVID-19 vaccination should be noted by eye care specialists, especially those that are vision-threatening for individuals with a history of allergic or autoimmune reactions. Doctors must provide intense care and attention to patients complaining of frequent headaches, blurred vision, or eye infections after the COVID-19 vaccination. These occult complications have relied on a temporal association, which does not establish causality. Future studies will be required to determine whether there is any connection between COVID-19 vaccinations of different types and ocular problems. If a patient experiences ocular pain and a change in vision following COVID-19 immunization, ophthalmologists and primary care physicians should be aware of this potential consequence. The ocular complications should be considered in future vaccine safety studies to limit the occurrence of vaccine-related ocular complications. More reports and clinical data are required in order to develop improved guidelines and insights due to the relatively small number of reports per specific phenomenon. The introduction of vaccines should not be discouraged by these similar studies, but producers and independent agencies should continue to monitor the changing data before drawing firm conclusions.

## Figures and Tables

**Table 1:** A summary of the published data concerning patients with ocular complications after COVID-19 vaccines.

N	Author/Study Country	Type of Study	Population/Cases Number	Age/Sex	Vaccine	Time from Vaccines to Complications Occurrence	Ocular Complication
1	(Yu, Ritterband, & Mehta, 2022) [6] Kyoto/Japan	Case Report	1	51/M	Moderna mRNA	Three days after immunization	Full-thickness corneal graft rejection with corneal edema, leading to transplant failure.
2	(Yeo, Kim, Lee, Yi, & Chung, 2023) [7] Suwon, Korea.	Retrospective	26	The mean age is 57 y range (27–76) M&F	either mRNA or adenoviral vector vaccines	Three weeks after vaccination	Thirty RVO cases, 14 RAO cases, and one case presented bilateral AMN and PAMM after the COVID-19 vaccine.
3	(Duran, & Aykac, 2023) [8] France	Case Report	1	30/F	a single dose of BNT162b2 mRNA (Pfizer-BioNTech)	One year ago,	Optic neuritis (ON)
4	(Roy, Chandra, Roy, & Shrotriya, 2022) [9] India	Case Report	3	27/F 48/F 40/M	Covishield vaccine AstraZeneca	5–12 days after vaccination	Optic Neuritis (ON)
5	(Alvarez et al., 2021) [10] Birmingham/England	Observational. Cohort Study	55	Average age—45 years (range: 18–75) M&F	AstraZeneca, 38/55 Pfizer-BioNTech, 13/55 Sinovac, 4/55	Average: 18 days (range: 1–69)	Optic Neuritis (ON); in all cases, 55 patients were diagnosed with (ON) after the COVID-19 vaccination
6	(ElSheikh, Hasee, Eleiwa, & Elhusseiny, 2021) [11] (NA)	A Retrospective Case Reports	1	18/F	Sinopharm	Five days after the second dose of the Sinopharm	Bilateral anterior uveitis
7	Younus & Mulla (2022) [12] Glasgow, United State	Case Report	1	78/F	COVID-19 vaccine	after the second dose.	Posterior Scleritis
8	(Testi et al., 2022) [13] London	A Multinational Case Series	70	The mean age was 51 years (range, 19–84 years)	Pfizer 40/ Astra-Zeneca, 17/Moderna10/ Sinopharm2, and Covaxin1	14 days following the COVID-19 vaccination The mean time to event was five days and six days (range, 1–14 days) after the first and second doses of the vaccine, respectively	ocular inflammatory event Anterior uveitis (*n* = 41, 58.6%), posterior uveitis (*n* = 9, 12.9%), and scleritis (*n* = 7, 10.0%)
9	(Bolletta et al., 2021) [14] Italy	Retrospective Study	34 14 M 20 F	a mean age of 49.8 years (range 18–83 years)	Pfizer23/ Astra-Zeneca7/, Moderna3/, Janseen (Ad26.COV2)1.	(Range 1–30 days).	Uveitis and other ocular complications. The cases were (3) herpetic keratitis, (2) anterior scleritis, (5) anterior uveitis (AU), (3) toxoplasma retinochoroiditis, (2) Vogt-Koyanagi-Harada (VKH) disease reactivations, (2) pars planitis, (2) retinal vasculitis, (1) bilateral panuveitis in new-onset Behçet’s disease, (3) multiple, evanescent white dot syndromes (MEWDS), (1) acute macular neuroretinopathy (AMN), (5) retinal vein occlusions (RVO), (1) non-arteritic ischemic optic neuropathy (NAION), (3) activations of quiescent choroidal neovascularization (CNV) secondary due to myopia or uveitis, and (1) central serous chorioretinopathy (CSCR)
10	(Cohen et al., 2022) [15] Israel	A Retrospective Study	24 (a cohort of 5 patients and 19 literature cases)	NA	Pfizer BioNTech BNT162b2mRNA	28 days post-vaccination	Herpetic ocular infections
11	(Pang, Pan, Guo, & Wu, 2022) [16] China	Case Report	9 7 F 2 M	The mean (SD) age was 44.7 ± 16.5 years (range, 19–78 years)	inactivated COVID-19 vaccine.	Average 7.1 days (within a range of 1–14 days). post-vaccination	4 cases of keratitis, 1 case of choroiditis, 1 case of uveitis, 1 case of scleritis, 1 case of acute retinal necrosis, and 1 case of iridocyclitis
12	(Amin et al., 2022) [17] Bangladesh	Case Report	1	41/M	a live virus vector (COVISHIELD vaccine-AstraZeneca)	average five months	retinal hemorrhage.
13	(Rallis, et al., 2022) [18] (UK)	Retrospective Analysis	10	NA	Pfizer Astra-Zeneca, Moderna, Sinopharm, and Covaxin	Twenty-eight days after the first dose of the COVID-19 vaccination.	Herpetic Eye Disease
14	Crnej, Khoueir, Cherfan, & Saad, 2021) [19] (NA)	Case Reports	1	71/M	BNT162b2 mRNA (BioNTech/Pfizer)	seven days after the first vaccination dosage.	Corneal endothelial transplant rejection
15	(Khan et al., 2021) [20] (NA)	Case Report	1	48/M	AstraZeneca	Five weeks following the initial dosage	Bilateral immune-mediated corneal melting and necrosis
16	(Penbe, 2022) [21] Turkey	Case Report	1	67/M	inactive vaccine for SARS-CoV-2	NA	Peripheral ulcerative keratitis (PUK) with nodular scleritis
17	Farrell, Deacon, & Mauger, 2022) [22] (USA)	Case Report	1	68/F	Moderna	2.5 weeks after the first dose	Marginal keratitis
18	(Lee & Han, 2022) [23] Korea	Case Report	1	55/F	AstraZeneca	Six days after	Bilateral transient corneal edema
19	Cunha et al., 2022) [24] Brazil	Case Report	1	50/M	Pfizer-BioNTech	Two weeks after	Recurrent transitory monocular visual loss attacks
20	Lee, Wu, Cheng, & Chang, 2022) [25] Taiwan	Case Report	1	41/F	Vaxzevria, AstraZeneca	Two days after	Central serous chorioretinopathy with disc edema in the other eye

M: Male. F: Female. RAO: retinal artery occlusion. RVO: retinal vein occlusion. AMN: acute macular neuroretinopathy. PAMM: Paracentral acute middle maculopathy. NA: Information Not Available. UK: United Kingdom.

**Table 2:** Distribution of the ocular Complications post-COVID-19 vaccines.

Ocular Complication	Percent	Number
Ocular inflammatory complications include “uveitis, anterior uveitis, posterior uveitis, scleritis, posterior scleritis, choroiditis, keratitis, acute retinal necrosis, and iridocyclitis”	(47.3%)	115
“Optic neuritis”	(24.3%)	59
“Herpetic eye disease”	(14%)	34
Retinal artery occlusion, retinal vein occlusion, acute macular neuroretinopathy, paracentral acute middle maculopathy	(10.7%)	26
Corneal endothelial transplant rejection	(0.4%)	1
Full-thickness corneal graft rejection leads to transplant failure	(0.4%)	1
Retinal hemorrhage.	(0.4%)	1
Bilateral immune-mediated corneal melting and necrosis	(0.4%)	1
Marginal keratitis	(0.4%)	1
Peripheral ulcerative keratitis (PUK) with nodular scleritis	(0.4%)	1
Bilateral transient corneal edema	(0.4%)	1
Recurrent transitory monocular visual loss attacks	(0.4%)	1
Central serous chorioretinopathy with disc edema in the other eye	(0.4%)	1

**Table 3:** The most common ocular complications categorization according to the type of vaccine given.

Ocular Complications	Vaccine Type	Cases Number (Total = 243)	Cases Percent %
Ocular inflammatory complications “Uveitis, Anterior uveitis, posterior uveitis, scleritis, Posterior Scleritis, Choroiditis, keratitis acute retinal necrosis and iridocyclitis”	Pfizer Astrazeneca, Moderna (Ad26.COV2) Sinopharm Covaxin Janssen Inactivated COVID-19 vaccine.	63 24 13 9 3 1 1 1	25.9% 9.9% 5.3% 3.7% 1.2% 0.41% 0.41% 0.41%
“Optic Neuritis”	AstraZeneca Pfizer Sinovac	41 14 4	16.9% 5.8% 1.6%
Retinal artery occlusion, Retinal vein occlusion, acute macular neuroretinopathy, and Paracentral acute middle maculopathy	mRNA and adenoviral vector vaccines	26	10.7%
“Herpetic eye disease”	Pfizer Pfizer, Astra-Zeneca, Moderna, Sinopharm, and Covaxin	24 10	9.9% 4.1%
Corneal endothelial transplant rejection	Pfizer	1	0.41%
Full-thickness corneal graft rejection leads to transplant failure	Moderna	1	0.41%
Retinal hemorrhage	AstraZeneca	1	0.41%
Bilateral immune-mediated corneal melting and necrosis	AstraZeneca	1	0.41%
Marginal keratitis	Moderna	1	0.41%
Peripheral ulcerative keratitis (PUK) with nodular scleritis	inactive vaccine	1	0.41%
Bilateral transient corneal edema	AstraZeneca	1	0.41%
Recurrent transitory monocular visual loss attacks	Pfizer	1	0.41%
Central serous chorioretinopathy with disc edema in the other eye	AstraZeneca	1	0.41%

## Data Availability

Data are available upon request from the researchers.
